# Complete mitochondrial genome and the phylogenetic position of the blackmouth croaker *Atrobucca nibe* (Perciformes: Sciaenidae)

**DOI:** 10.1080/23802359.2018.1456368

**Published:** 2018-04-01

**Authors:** Wei-Di Yang, Chang-Chang Guo, Min Liu, Baian Lin

**Affiliations:** College of Ocean and Earth Sciences, Xiamen University, Xiamen City, Fujian Province, China

**Keywords:** *Atrobucca*, Bayesian tree, mitogenomes, phylogenetic relationship, Sciaenidae

## Abstract

In this study, the complete mitogenome of the blackmouth croaker *Atrobucca nibe* was obtained. Its mitogenome is 16,842 bp in length, consisting of 37 genes with the typical gene order and direction of transcription in vertebrates. The overall nucleotide composition is: 27.2% A; 31.2% C; 16.4% G and 25.2% T. Sizes of the 22 tRNA genes range from 66 to 74 bp. One start codons (ATG) and three stop codons (TAG, AGA, and TAA/TA/T) were detected in 13 protein-coding genes. In the Bayesian tree based on the complete mitogenomes of 20 species (including *A. nibe*) from the family Sciaenidae, all nodes were strongly supported. The result suggested that *A. nibe* was subsequent to the group with genera *Otolithes*, *Chrysochir*, *Megalonibea*, *Pennahia*, *Nibea*, *Dendrophysa*, and *Johnius*.

The family Sciaenidae (Perciformes) is commonly known as croakers and drums. As a commercially important group of fishes, the family comprises approximately 270 species in 70 genera in the world (Nelson 2006). The genus *Atrobucca* consists of 10, small to medium sized, species, and the blackmouth croaker *Atrobucca nibe* (Jordan and Thompson, 1911) occurs in Indo-West Pacific and inhabits along coastal waters from 45 to 200 m depth (http://www.fishbase.org). In this study, we presented the complete mitochondrial genome of *A. nibe* and analysed its phylogenetic relationship based on another 19 available mitogenomes in Sciaenidae using one available mitogenome in the family Epinephelidae and two in the family Polynemidae as an outgroup.

One specimen of *A. nibe* was collected by a bottom trawler in the coastal water of Wenling County, Zhejiang Province, China. The protocol and data analysis methods followed Chen et al. (2014). The complete mitochondrial genome of *A. nibe* is 16,842 bp in length (GenBank accession number: MF004314) with the typical gene order and transcriptional direction in vertebrates. It contains two rRNA genes, 22 tRNA genes, 13 protein-coding genes, and one control region. The overall nucleotide composition is as follows: 27.2% A; 31.2% C; 16.4% G and 25.2% T. In the 13 protein-coding genes, only one start codon (ATG) was detected. Three stop codons (TAG, AGA, and TAA/TA/A) were found; *ND1* was terminated by the TAG codon, *COX1* by the AGA codon, and the other 11 protein-coding genes by either the TAA or incomplete T or TA codon that may form the complete termination signal UAA via post-transcriptional polyadenylation (Ojala et al. 1981). The 12S (953 bp) and 16S (1699 bp) rRNA genes are located between the tRNA-*Phe* and tRNA-*Leu1* genes, separated by the tRNA-*Val* gene. The lengths of 22 tRNA genes range from 66 to 74 bp; 21 tRNAs can be folded into the typical cloverleaf secondary structures with the exception of tRNA-*Ser2* in which the DHU arm was replaced by a simple loop. A 40 bp inserted sequence was identified as the putative origin of L-strand replication (OL). The control region was 1159 bp in length with high A + T (65.3%) and low G + C (34.7%) composition.

Published mitogenomes of all 20 species from Sciaenidae (including *A. nibe* in this study) together with *Cephalopholis boenak* from Epinephelidae, and *Eleutheronema tetradactylum* and *Polydactylus sextarius* from Polynemidae were used to assess the phylogenetic relationship of *A. nibe*. Phylogenetic tree was constructed with the partitioned Bayesian method based on the dataset combined by three partitions (the alignments of the 1, 2 codon positions of 12 H-strand encoded protein-coding genes together with 12S and 16S rRNA genes) under the GRT + I+G model (Ronquist and Huelsenbeck 2003). As the phylogenetic tree showed, all nodes were strongly supported with high value of posterior probability (Figure 1). The result suggested that *A. nibe* was subsequent to the group with genera *Otolithes*, *Chrysochir*, *Megalonibea*, *Pennahia*, *Nibea*, *Dendrophysa*, and *Johnius* which is consistent to the croaker phylogenetic results using combined mitochondrial and nuclear genomes (Lo et al. 2015).

**Figure 1.**
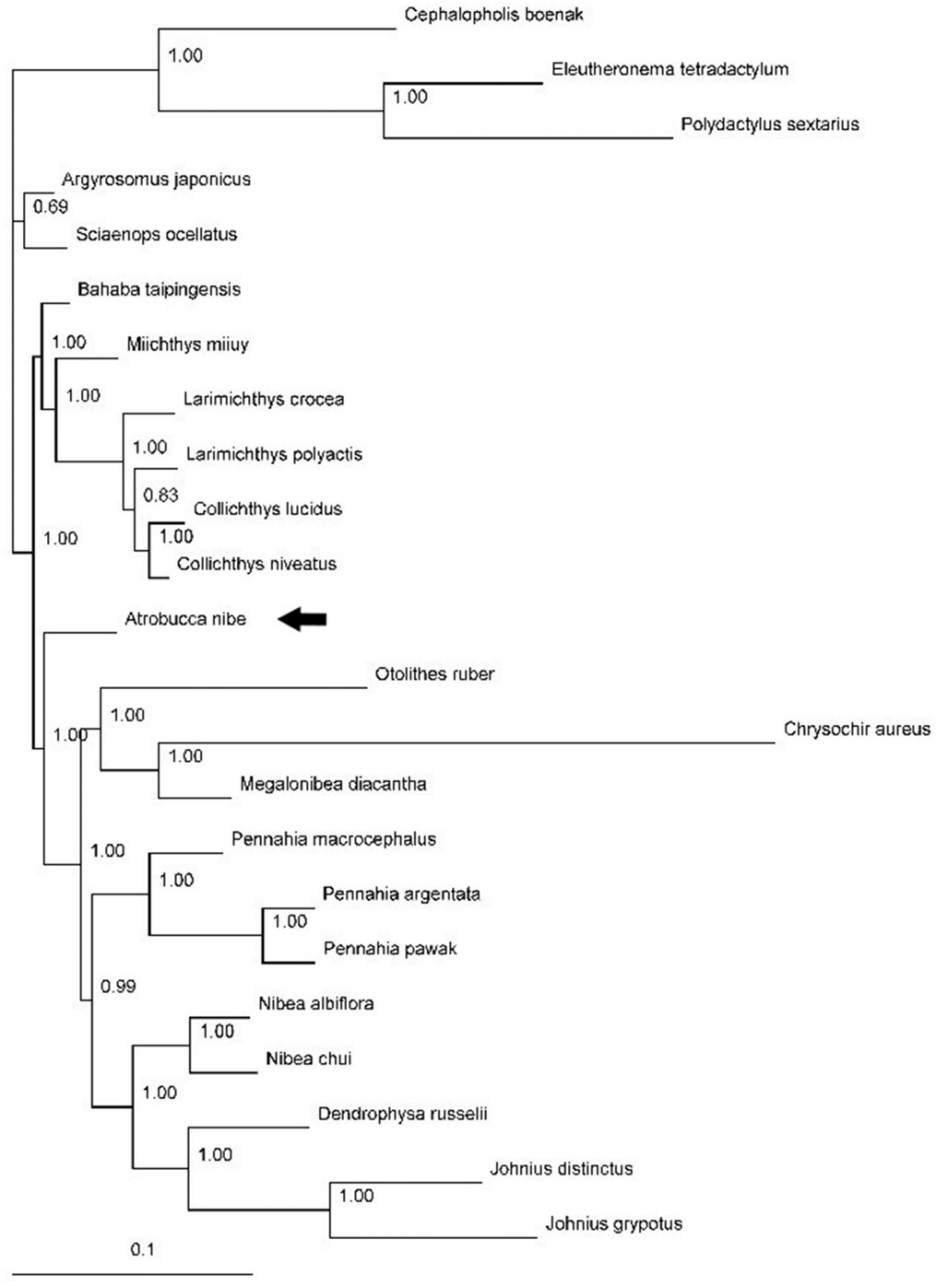
Phylogenetic position of the blackmouth croaker *A. nibe* (MF004314). *Cephalopholis boenak* (KC537759), *Eleutheronema tetradactylum* (KC878730), and *Polydactylus sextarius* (NC_027088) were selected as the outgroup. The other 19 species from Sciaenidae are: *Argyrosomus japonicus* (KT184692), *Bahaba taipingensis* (NC_018347), *Chrysochir aureus* (MF004313), *Collichthys lucidus* (JN857362), *Collichthys niveatus* (JN678726), *Dendrophysa russelii* (JQ728562), *Johnius distinctus* (MF083699), *Johnius grypotus* (KC491206), *Larimichthys crocea* (NC_011710), *Larimichthys polyactis* (GU586227), *Megalonibea diacantha* (KM257722), *Miichthys miiuy* (NC_014351), *Nibea albiflora* (NC_015205), *Nibea chui* (NC_025307), *Otolithes ruber* (KX929060), *Pennahia argentata* (NC_015202), *Pennahia macrocephalus* (KX576460), *Pennahia pawak* (KY978753), and *Sciaenops ocellatus* (NC_016867).
